# A Case Report on Sternal Tuberculosis Osteomyelitis: From Diagnosis to Recovery

**DOI:** 10.7759/cureus.72514

**Published:** 2024-10-28

**Authors:** Leena Saeed, Hanna Elbashir, Tibyan Y Abdalhamed, Majda Shanta, Zeinab Alsiddig A Ibrahim, Layla M Mahir

**Affiliations:** 1 Medical Research Center, Hamad Medical Corporation, Doha, QAT; 2 Emergency Medicine, Hamad Medical Corporation, Doha, QAT; 3 Internal Medicine, Hamad Medical Corporation, Doha, QAT; 4 General Medicine, Phi MedCare, Doha, QAT; 5 Radiology, Hamad Medical Corporation, Doha, QAT

**Keywords:** anti-tuberculosis therapy, osteomyelitis, sternal tuberculosis, tb osteomyelitis, tuberclusis

## Abstract

Sternal tuberculosis (TB) is a rare extrapulmonary manifestation of TB that usually manifests with nonspecific symptoms. Timely diagnosis and intervention are essential to avoid complications. Here, we discuss a case involving a 46-year-old male who experienced right scapular pain for six months and sternal swelling for 11 months. Although initial tests were negative, further investigations ultimately confirmed the diagnosis of sternal TB. The patient was successfully treated with anti-TB medications. Sternal TB remains a rare but important consideration in patients with unexplained chest wall swelling. Clinicians should be aware of this condition and its diagnostic challenges to ensure timely and appropriate management.

## Introduction

Tuberculosis (TB) caused 1.3 million deaths worldwide, highlighting its impact as a leading infectious disease. TB, a curable and preventable illness primarily affecting the lungs, presents approximately 10.6 million new cases annually [1]. It is caused by *Mycobacterium tuberculosis* and often begins as a latent infection, which can remain asymptomatic and undetected. However, when latent TB progresses to active disease, it commonly affects the lungs and becomes communicable. TB can also involve almost any other organ system, leading to extrapulmonary TB (EPTB) [2], including rare cases of sternal involvement.

Isolated sternal involvement in tuberculous osteomyelitis is rare, representing less than 1% of all cases, even in regions where TB is prevalent [3,4]. This rarity necessitates a high index of suspicion for diagnosing tuberculous osteitis in patients with undiagnosed bone lesions [5]. The diagnosis of sternal TB is particularly challenging due to its uncommon presentation and the infrequent nature of this form of the disease, often resulting in delays in recognition and treatment [6].

Recent advances in diagnostic techniques, such as rapid molecular testing and whole-genome sequencing, along with the WHO's recommendation for all-oral therapy for drug-resistant TB in 2018, offer potential improvements in managing this complex condition [7]. This case report aims to highlight the rare occurrence of tuberculous involvement of the sternum and underscore the importance of considering this diagnosis in atypical presentations.

## Case presentation

A 46-year-old Indian male laborer presented with a six-month history of pain in the right scapula and an 11-month history of swelling in the chest wall soft tissue. The patient reported that the swelling initially began as pain and subsequently developed into an open wound. Over time, the wound has gradually increased in size and is associated with pus discharge.

The mass was not associated with weight loss or hemoptysis, and other systematic reviews yielded negative results. The patient denied any family history of TB, recent travel to his home country, or household contact with TB. He is neither an alcoholic nor a smoker. During the initial visit, he was following up with orthopedics as an outpatient. Upon examination, vital signs were within normal limits. His recorded vital signs included a temperature of 37°C, blood pressure of 124/79 mmHg, pulse rate of 95 bpm, respiratory rate of 18 breaths per minute, and oxygen saturation of 100% on room air. The patient's body mass index was 25.97 kg/m².

Examination of the chest wall revealed a soft tissue mass located on the anterior chest wall midline over the sternum. The mass exhibited redness, warmth, and mild tenderness and was associated with severe pain at the lower border of the scapula. Initial laboratory tests were ordered by the orthopedics department, including acid-fast bacillus (AFB) smear and culture, TB polymerase chain reaction (PCR), CBC, human immunodeficiency virus (HIV) test, and hepatitis B virus test; all demonstrated no significant findings (Table 1).

**Table 1:** Initial laboratory tests WBC: White blood cell; ANC: absolute neutrophil count; ALK: alkaline phosphatase; ALT: alanine transaminase; AST: aspartate transaminase; CRP: C-reactive protein; HBV: hepatitis B virus; HIV: human immunodeficiency virus; PCR: polymerase chain reaction

Test	Result	Normal range
WBC	11.3	4-10 x 10^3^/uL
ANC	8.3	2-7 x 10^3^/uL
Urea	5.6	2.5-7.8 mmol/L
Creatinine	69	62-106 umol/L
ALK	125	40-129 U/L
ALT	94	0-41 U/L
AST	51	0-40 U/L
CRP	7.2	0-5
HBV antigen	Negative	
HIV	Negative	
QuantiFERON TB PCR	Negative	
Acid-fast bacilli	Negative	

**Figure 1:**
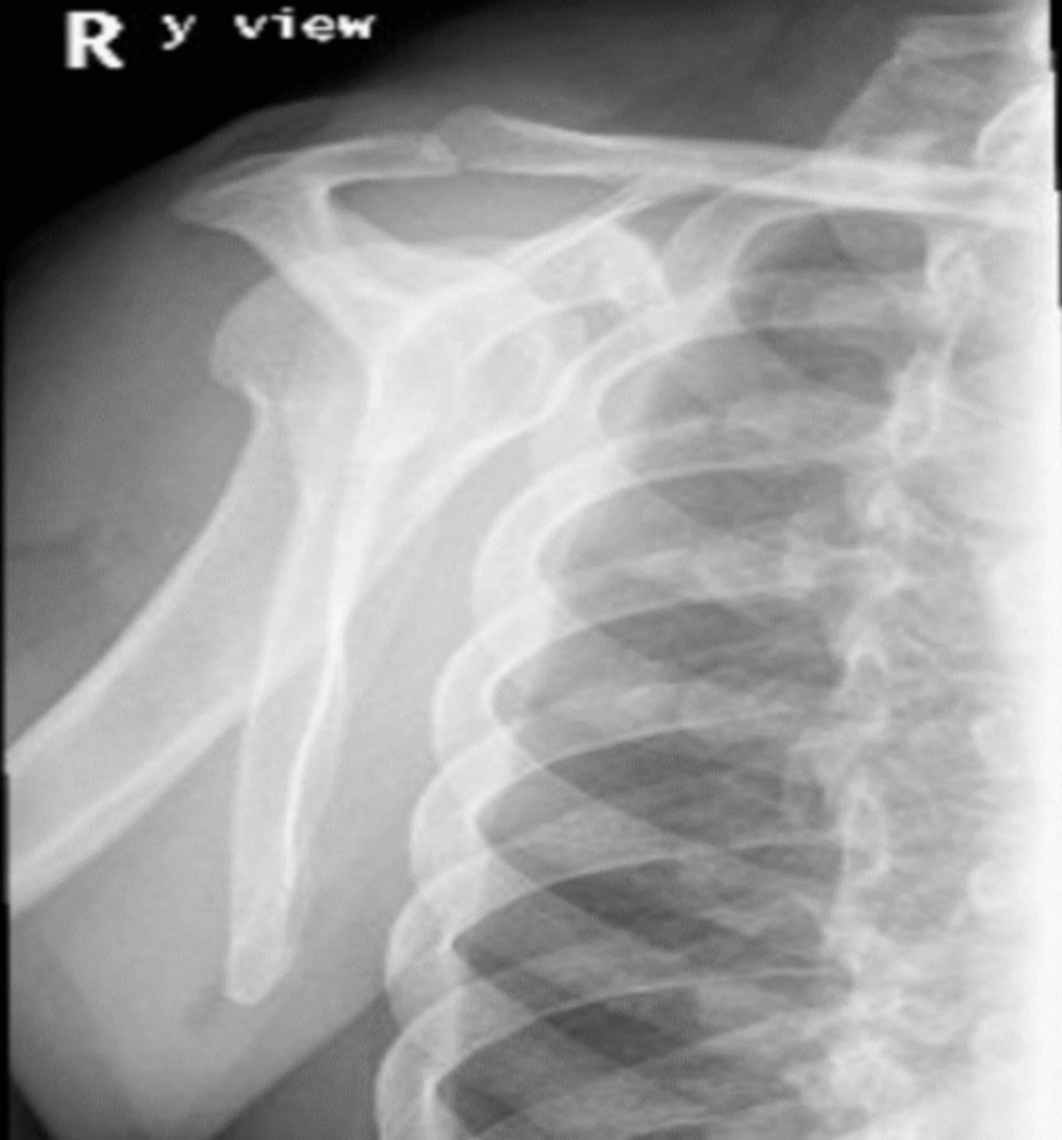
X-ray of the scapula

**Figure 2:**
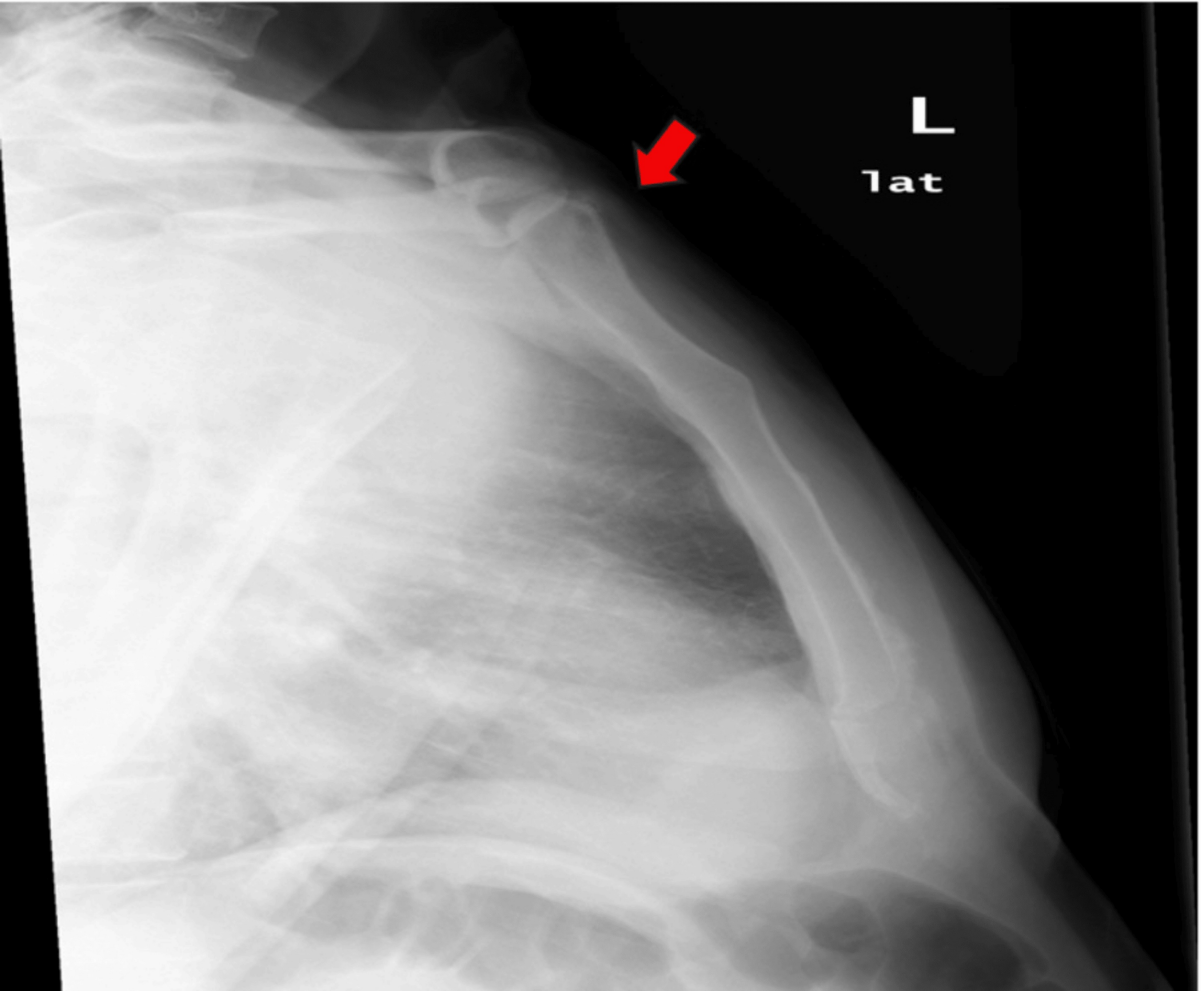
There is lytic lesion in the anterior superior aspect of the sternum with surrounding increased sclerosis (red arrow)

**Figure 3:**
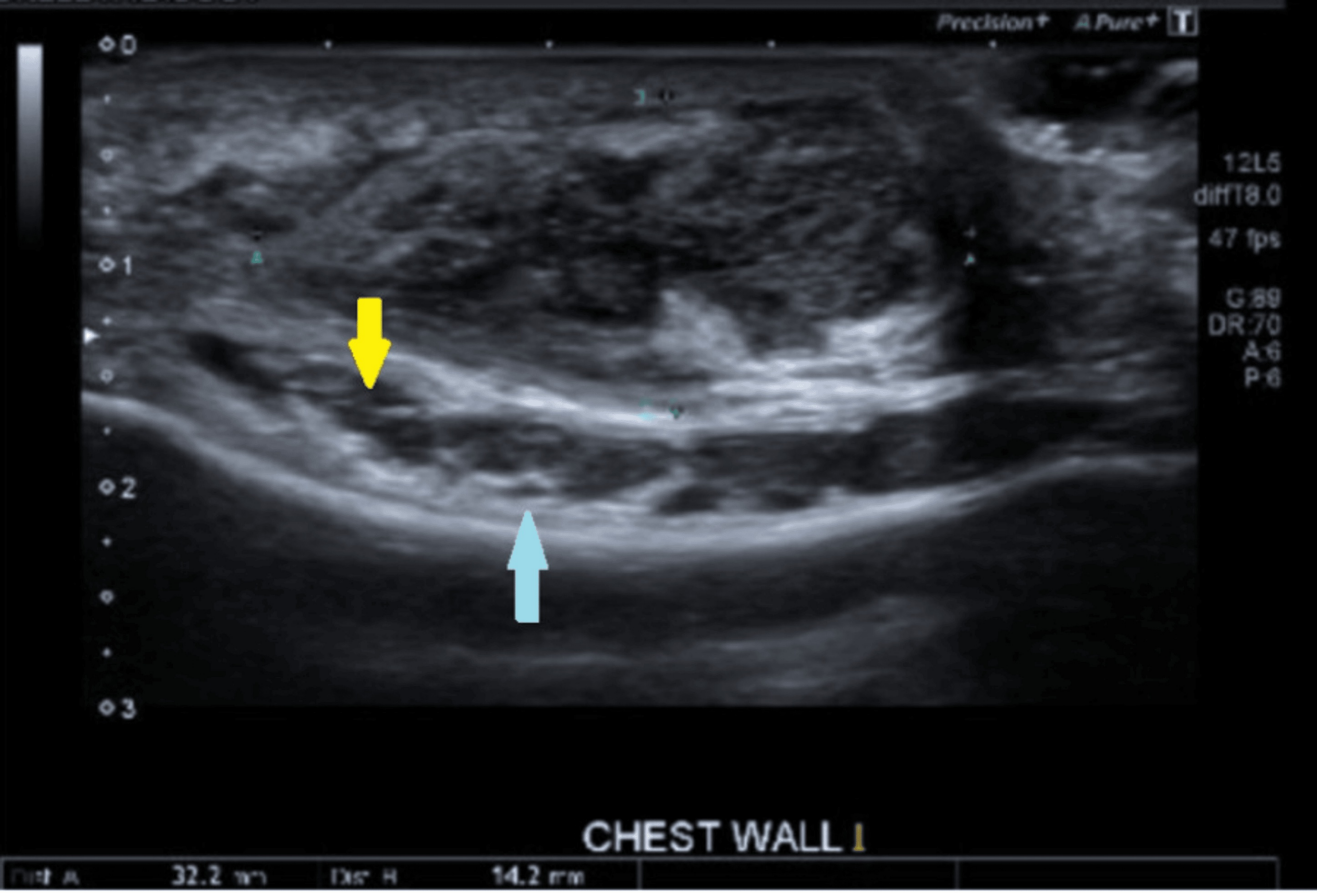
An ultrasound of the chest wall showed small collection 32 x 14 mm on the upper sternum (yellow arrow) with bony irregularity (blue arrow)

**Figure 4:**
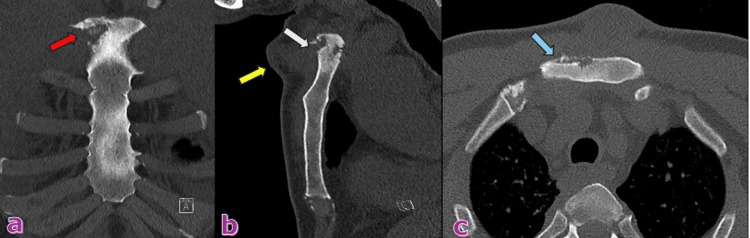
There is an ill-defined mixed sclerotic and lytic appearance of the right superolateral aspect of the manubrium (red arrow) seen in the coronal plane (a). The lesion appears near the right first costo-sternal joint (blue arrow) in the axial cut (c). There is anterior and posterior areas of cortical breakage (white arrow) with surrounding soft tissue swelling (yellow arrow) in the sagittal plane (b)

An X-ray of scapula showed no fractures (Figure 1), but X-ray of sternum revealed a lytic lesion in the anterior superior aspect of the sternum with surrounding increased sclerosis (Figure 2). On the same day, an ultrasound and a CT scan were performed. The ultrasound findings were suggestive of osteomyelitis (Figure 3). The ultrasound findings were suggestive of osteomyelitis (Figure 3). The CT scan confirmed osteomyelitis (Figure 4) and indicated that potential causes and differential diagnosis such as malignancy need to be excluded.

**Figure 5:**
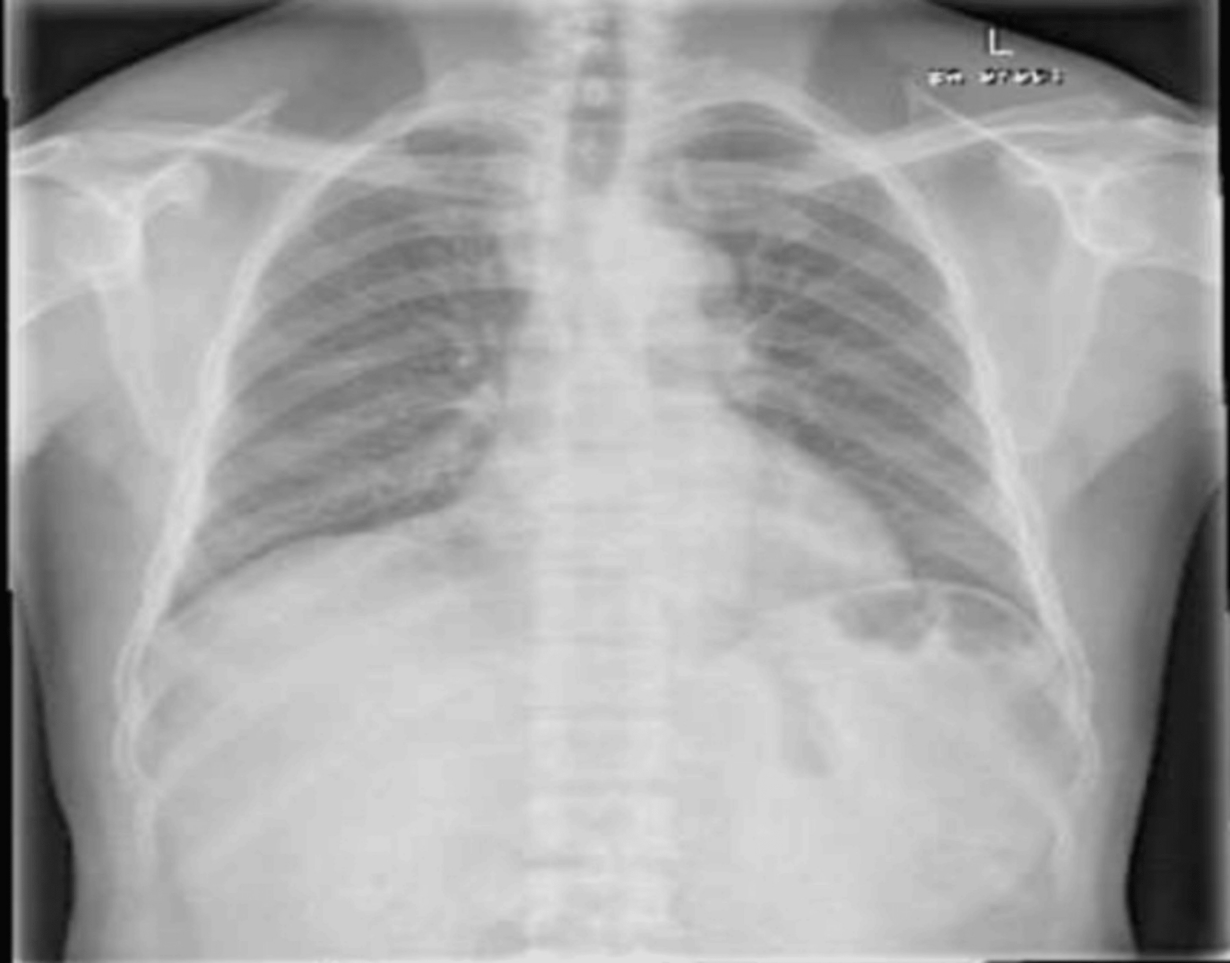
The cardiothoracic ratio is normal. Both lungs are well-expanded and clear, with clear costophrenic angles

During the patient's second visit to orthopedics, a chest X-ray (Figure 5) and an X-ray of the thoracolumbar spine were ordered, both of which returned normal. A repeat AFB smear and culture, along with TB PCR testing, were performed. The QuantiFERON test (QFT) returned positive, and acid-fast bacilli were identified on smear and culture from the aspiration sample. Additionally, a pus culture from the wound showed no growth of organisms (Table 2). The diagnosis of TB of the manubrium sternum was confirmed. The patient was subsequently referred to the infectious disease department for treatment and was started on a regimen of rifampin/isoniazid 30 mg/150 mg, taking two tablets daily.

**Table 2:** Additional laboratory tests WBC: White blood test; ALK: alkaline phosphatase; ALT: alanine transaminase; AST: aspartate transaminase; TB: tuberculosis; PCR: polymerase chain reaction; MTB: mycobacterium tuberculosis

Test	Result	Normal tange
WBC	10.0	4-10 x 10^3^/uL
ALK	159	40-129 U/L
ALT	159	0-41 U/L
AST	54	0-40 U/L
Acid-fast bacilli	Positive	
TB PCR	Positive	
TB MTB culture	Positive for Mycobacterium organism	
TB PCR rifampicin resistance	Indeterminate	
Pus culture	Negative for organism	

Two days after starting treatment, the patient exhibited elevated liver function tests. As a result, anti-TB medications were held to assess the severity of the liver injury, and swabs were taken for TB testing and microbiological culture. The aspirated pus culture tested positive for AFB PCR, with rifampin resistance reported as indeterminate (Table 2). Additionally, an MRI of the sternum and an abdominal ultrasound were requested. The patient was referred for follow-up with thoracic surgery.

**Figure 6:**
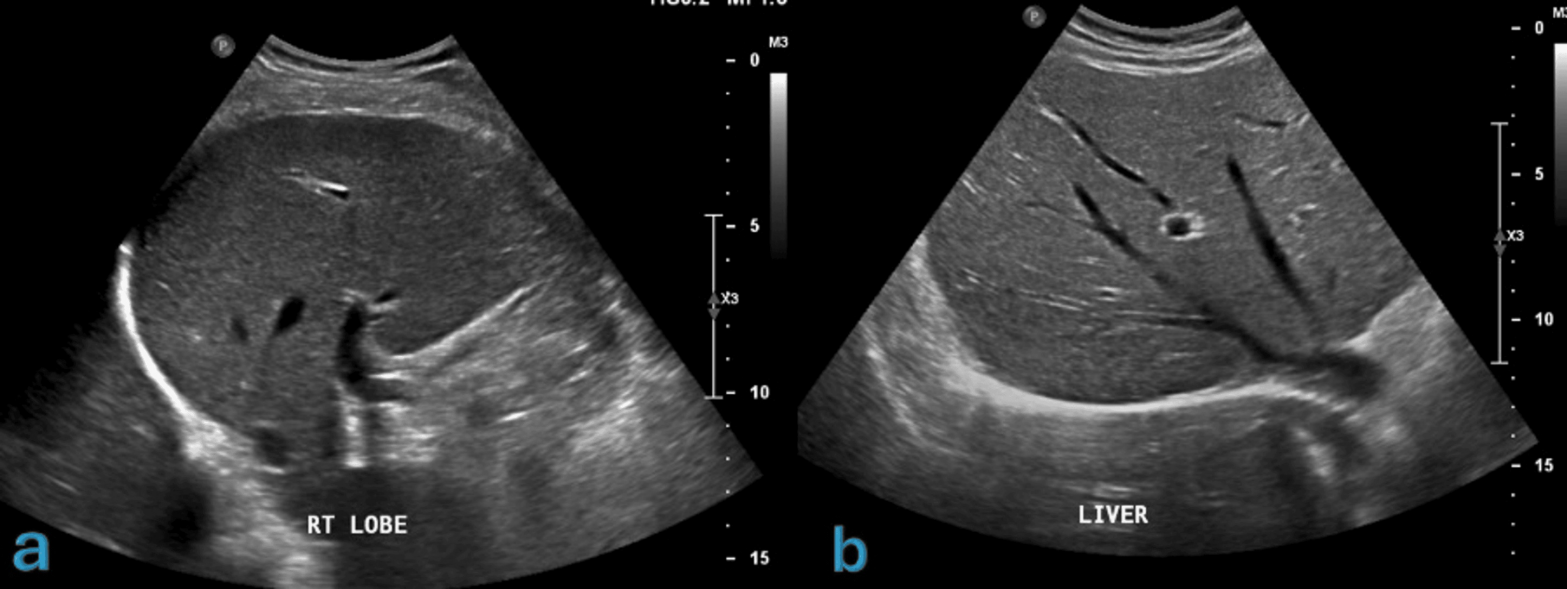
Right hepatic lobe demonstrating slight coarse hepatic parenchymal echotexture with slight prominent periportal echogenicity may suggest periportal fibrosis (PPF)

**Figure 7:**
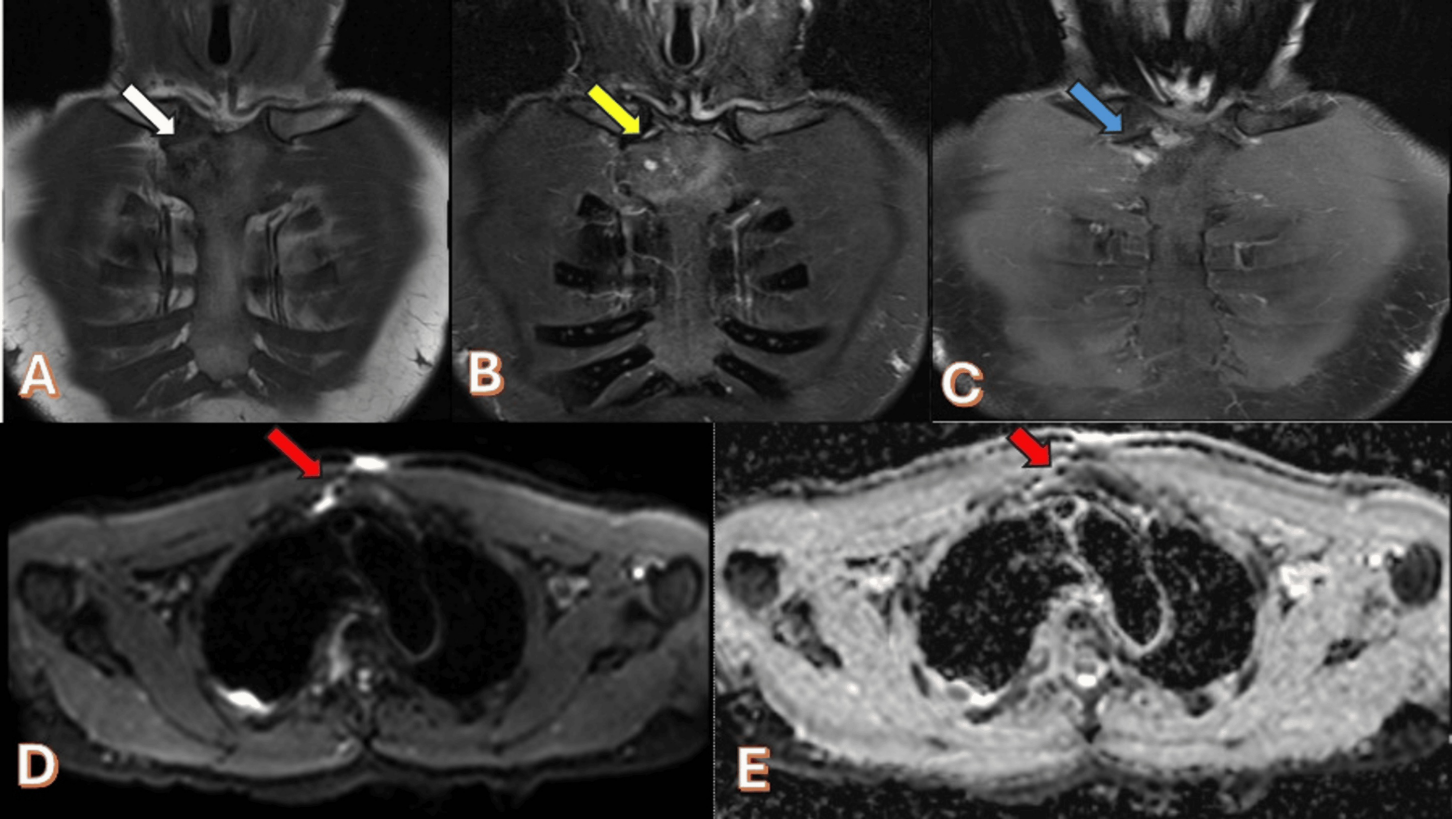
(A) Coronal precontract T1WI shows the following: Ill-defined iso- to hypointense lesion seen in the right lateral aspect of the manubrium (white arrow) demonstrating heterogenous predominately high signal intensity seen in coronal T2WI (yellow arrow) (B). The lesion shows post-contrast enhancement noted in T1 coronal fat sat post-contrast image (blue arrow) (C). There is an area of diffusion restriction noted as high signal in DWI and low signal in ADC in (D) and (E), respectively (red arrow) T1WI: T1-weighted Image; T2WI: T2-weighted Image; DWI: diffusion-weighted image; ADC: apparent diffusion coefficient

Abdominal ultrasound was done (Figure 6), along with MRI of the sternum which revealed osteomyelitis with a small, localized subcutaneous pyogenic collection (Figure 7). Subsequently, the patient started presumptive anti-TB treatment, consisting of Rifafour and pyridoxine 40 mg. The patient tolerated the medications well, with no reported complications.

**Figure 8:**
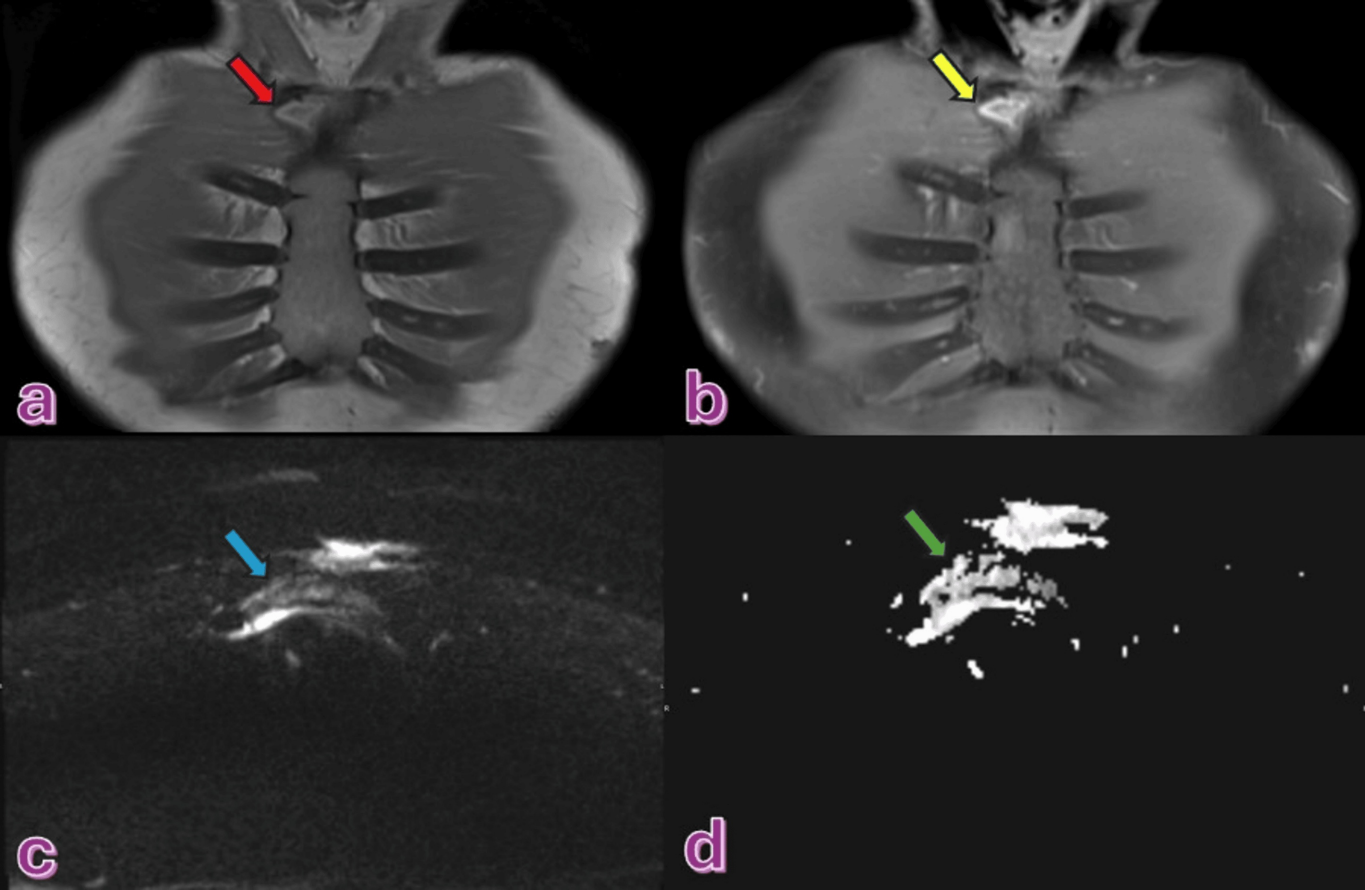
A well-defined slightly heterogenous lesion with intermediate signal noted in the lateral right aspect of the manubrium sternum in T1WI (red arrow) (a), the lesion demonstrates post-contrast enhancement seen in the post-contrast T1W1 (yellow arrow) (b). It also shows partial diffusion restriction in DWI (c) and ADC (d) (blue and green arrow)

Two months later, the patient was under follow-up with the cardiothoracic surgery team, who requested an MRI of the sternum to assess the progress. The MRI revealed areas of abnormal signal intensity (Figure 8). Based on the MRI results, the decision was made to extend the treatment duration to 9-12 months.

**Figure 9:**
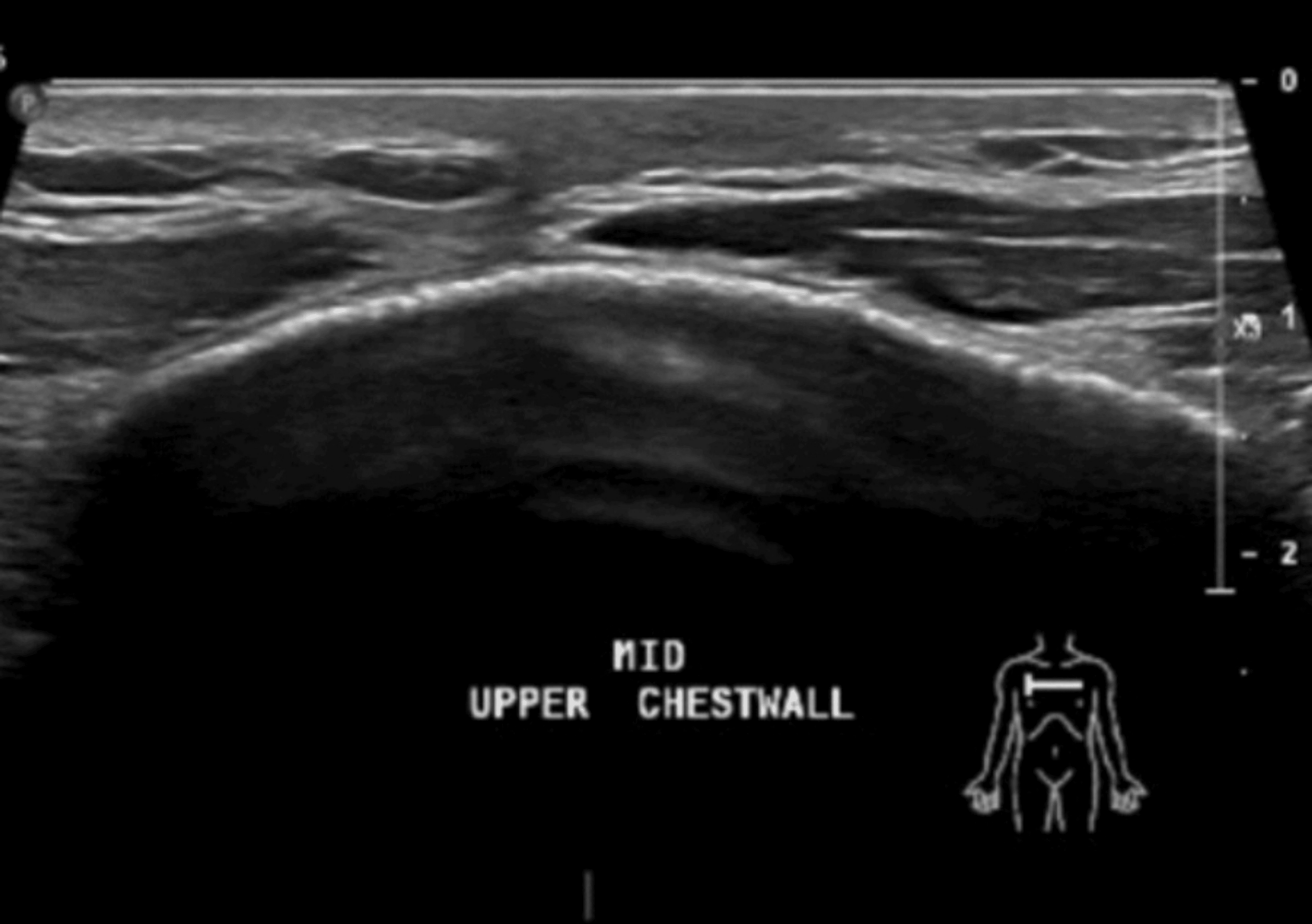
Normal chest wall ultrasound

The patient completed a total of 12 months of treatment for TB osteomyelitis. A follow-up chest wall ultrasound was performed to assess any residual collections. The ultrasound revealed no obvious or sizeable collections (Figure 9).

## Discussion

"The Great Imitator" is often used to describe medical conditions that present with vague or nonspecific symptoms, making them easily mistaken for various other diseases. Several infectious diseases have earned this designation, such as TB, though syphilis is the oldest and most renowned example [8].

In this case report, we describe a 46-year-old male with an 11-month history of swelling in the sternal region. Following extensive diagnostic evaluation, the patient was ultimately diagnosed with sternal TB. The definitive diagnosis of tuberculous sternal infection is challenging due to the condition's rarity and the absence of distinct signs and symptoms typically associated with TB [9].

TB of the sternum is an uncommon manifestation of flat-bone TB, which can present either as an isolated condition or in conjunction with pulmonary or lymphatic involvement [10]. As such, a chest X-ray is recommended to assess for pulmonary TB, and an abdominal ultrasound should be performed to evaluate potential lymph node involvement.

TB of the chest wall can resemble bone lymphoma due to its gradual progression. However, patients often present with a history of weight loss, night sweats, and pruritus, along with an elevated erythrocyte sedimentation rate (ESR) [11]. The presence of lumps often raises concerns about tumors. Consequently, a thorough diagnostic evaluation and imaging are essential to exclude other possible pathologies.

The diagnosis of sternal TB infections primarily relies on timely microbiological investigations, with culture results being more dependable than medical imaging. Among patients with EPTB, false-negative QFT results occurred in 28.8% of cases. The specific site of TB may impact the positive detection rate of QFT. Consequently, it is crucial to consider the possibility of TB, particularly in patients showing clinical signs of pleural or bone involvement, even when their QFT results are negative [12]. Among imaging techniques, CT and MRI are more informative than chest X-rays for potential diagnosis. MRI is effective in identifying bone and soft-tissue edema [13]. A typical feature of TB osteomyelitis on MRI is the increased intensity of normal bone marrow on T2-weighted imaging [14].

A high index of suspicion is essential for the early diagnosis of sternal TB, allowing for prompt initiation of treatment to prevent complications. The need for surgical intervention in the management of sternal TB has not yet been conclusively determined [13,15].

## Conclusions

In conclusion, TB of the sternum is a rare manifestation of osteomyelitis, accounting for less than 1% of cases. While TB osteomyelitis more commonly affects the spine, small bones of the hands and feet, femur, and tibia, clinicians must maintain a high index of suspicion when encountering atypical presentations such as sternal involvement.

Early diagnosis requires thorough clinical evaluation, laboratory workup, and extensive imaging to exclude other differential diagnoses. Prompt initiation of anti-tubercular therapy is crucial to ensure recovery and prevent severe complications, such as extensive bone destruction and deformity.
